# Comparative mitogenome analysis of two ectomycorrhizal fungi (*Paxillus*) reveals gene rearrangement, intron dynamics, and phylogeny of basidiomycetes

**DOI:** 10.1186/s43008-020-00038-8

**Published:** 2020-07-02

**Authors:** Qiang Li, Yuanhang Ren, Dabing Xiang, Xiaodong Shi, Jianglin Zhao, Lianxin Peng, Gang Zhao

**Affiliations:** 1 Key Laboratory of Coarse Cereal Processing, Ministry of Agriculture and Rural Affairs, College of Pharmacy and Biological Engineering, Chengdu University, Chengdu, 610106 Sichuan China; 2 Present address: Key Laboratory of Coarse Cereal Processing, Ministry of Agriculture and Rural Affairs, 2025 # Chengluo Avenue, Chengdu, 610106 Sichuan China

**Keywords:** *Basidiomycota*, *Boletales*, Ectomycorrhizas, Gene rearrangement, Mitochondrial genome, Repeat

## Abstract

In this study, the mitogenomes of two *Paxillus* species were assembled, annotated and compared. The two mitogenomes of *Paxillus involutus* and *P. rubicundulus* comprised circular DNA molecules, with the size of 39,109 bp and 41,061 bp, respectively. Evolutionary analysis revealed that the *nad4L* gene had undergone strong positive selection in the two *Paxillus* species. In addition, 10.64 and 36.50% of the repetitive sequences were detected in the mitogenomes of *P. involutus* and *P. rubicundulus,* respectively, which might transfer between mitochondrial and nuclear genomes. Large-scale gene rearrangements and frequent intron gain/loss events were detected in 61 basidiomycete species, which revealed large variations in mitochondrial organization and size in *Basidiomycota*. In addition, the insertion sites of the basidiomycete introns were found to have a base preference. Phylogenetic analysis of the combined mitochondrial gene set gave identical and well-supported tree topologies, indicating that mitochondrial genes were reliable molecular markers for analyzing the phylogenetic relationships of *Basidiomycota*. This study is the first report on the mitogenomes of *Paxillus*, which will promote a better understanding of their contrasted ecological strategies, molecular evolution and phylogeny of these important ectomycorrhizal fungi and related basidiomycete species.

## INTRODUCTION

Most boreal and north temperate forest tree species form mutualistic symbiotic relationships with ectomycorrhizal fungi (ECMF), which are of great significance to the balance and stability of forest ecosystems, carbon and nitrogen cycles, and the growth and reproduction of the fungi and host tree.

The genus *Paxillus* is an important ectomycorrhizal fungal group, widely distributed in the northern hemisphere. *Paxillus* species form ectomycorrhizal associations with various host plant species, including coniferous and hardwood trees (Hedh et al. 2009). It was reported that *Paxillus* can enhance the abiotic stress tolerance and promote the growth of host plants (Franco et al. 2014; Li et al. 2012; Ma et al. 2014). *Paxillus involutus*, the most studied species in the genus, has become one of the model organisms for revealing the evolution, life-style and physiology of ECMF (Rineau et al. 2012; Shah et al. 2013).

Studies of *Paxillus* nuclear genomes have found that species of this genus have a reduced complement of genes encoding plant cell wall-degrading enzymes (PCWDEs) and retain a unique array of PCWDEs to adapt to mutualistic symbiotic life-styles (Kohler et al. 2015). Some nuclear genes have also evolved to better adapt to such life-styles (Kohler et al. 2015; Rajashekar et al. 2007). However, up to now, their mitochondrial genetic information has not yet been elucidated, limiting our overall understanding of the genetics and evolution of *Paxillus* species.

The genus *Paxillus* was divided into two groups in Europe, the *P. involutus* complex and *P. rubicundulus*. *Paxillus rubicundulus* occurs mainly in wetland habitats and only forms a relationship with *Alnus* species (Jargeat et al. 2016), while the *P. involutus* complex occurs in more diverse habitats and forms mutualistic relationships with various tree species. Therefore, host specificity could be used as a useful feature to distinguish *P. rubicundulus* (Jargeat et al. 2016), but accurately identifying species in the *P. involutus* complex remains difficult (Jargeat et al. 2014). Phylogenetic studies have revealed four genetic lineages within the *P. involutus* complex (Jargeat et al. 2014), and in addition there are some species whose taxonomic status remains confusing. This presents difficulties for efficient utilization and research into those *Paxillus* species. Mitochondrial genes have been reported as a powerful tool for the study of phylogeny due to several advantages (Delsuc et al. 2003; Nie et al. 2019), but up to now no mitochondrial genome (mitogenome) from the genus *Paxillus*, or even the family *Paxillaceae*, has been available.

The mitogenome of eukaryotic organisms was reported to have been obtained from the common ancestral *Alphaproteobacteria* though endosymbiosis (Lang et al. 1999). In basidiomycete species, they generally contain 15 core protein coding genes (PCGs), including 14 genes for energy metabolism (*atp6*, *atp8*, *atp9*, *cob*, *cox1*, *cox2*, *cox3*, *nad1*, *nad2*, *nad3*, *nad4*, *nad4L*, *nad5*, and *nad6*) and 1 *rps3* gene for transcriptional regulation. In addition, 20–36 tRNA genes and 2 rRNA genes were also detected in basidiomycete mitogenomes (Li et al. 2018a; Li et al. 2018d). The mitogenome has become a powerful tool for the study of phylogeny due to several available molecular markers and uniparental inheritance (Andersen and Balding 2018; Wang et al. 2018). With the rapid development of next-generation sequencing technology, more and more nuclear and mitochondrial genomes of eukaryotic organisms have been obtained (Tajima et al. 2016). However, compared with their animal counterparts, the mitogenomes of fungi have been less studied (Sandor et al. 2018), especially that of basidiomycetes in the basidiomycete class *Agaricomycetes* (the largest mushroom-forming fungal group). The limited studies that have been carried out on fungal mitogenomes have, however, shown that these vary greatly in genome size, gene content, gene arrangement, intron number, and content of repeat sequences, even between closely related species (Li et al. 2018c; Li et al. 2019b).

In this study, the mitogenomes of two *Paxillus* species, *P. involutus* and *P. rubicundulus*, were assembled and annotated in order to: (1) reveal features of *Paxillus* mitogenomes and the variations or similarities between those of the two species in genome size, gene content, gene arrangement, and repeat sequence; (2) demonstrate any dynamic changes of introns and gene rearrangement in the two *Paxillus* mitogenomes compared with other basidiomycete mitogenomes to contribute to a more comprehensive understanding of their size and organization variation in basidiomycetes; and (3) analyze the phylogenetic relationships of *Basidiomycota* based on the combined mitochondrial gene set. The mitogenomes of the two *Paxillus* species could further our understanding of the taxonomy, evolution, and potential molecular mechanisms driving host specificity in an important ectomycorrhizal.

## MATERIALS AND METHODS

### Mitochondrial genome assembly and annotations

The raw sequencing data of *Paxillus involutus* and *P. rubicundulus* were downloaded from the Joint Genome Institute (JGI) database (Kohler et al. 2015). A series of quality control steps were conducted to obtain clean reads from the raw sequencing data. First, we removed adapter reads using AdapterRemoval v2 (Schubert et al. 2016), and then ngsShoRT (Chen et al. 2014) to filter low-quality sequences. The *Paxillus* mitogenomes were assembled with the resulting clean reads using SPAdes 3.9.0 (Bankevich et al. 2012), and gaps between contigs were filled using MITObim V1.9 (Hahn et al. 2013). The complete mitogenomes obtained were first annotated according to our previously described methods (Li et al. 2018a; Li et al. 2018d). That is, the protein-coding genes (PCGs), rRNA genes and tRNA genes of the mitogenomes were initially annotated using MFannot (Valach et al. 2014) and MITOS (Bernt et al. 2013), based on the genetic code 4. PCGs were then predicted or modified with the NCBI Open Reading Frame Finder (Coordinators 2017), and further annotated by BLASTP searches against the NCBI non-redundant protein sequence database (Bleasby and Wootton 1990). tRNA genes were also predicted and identified with tRNAscan-SE v1.3.1 (Lowe and Chan 2016). Graphical maps of the two complete mitogenomes were drawn with OGDraw v1.2 (Lohse et al. 2013).

### Sequence analysis of the *Paxillus* mitogenomes

Base compositions of the two *Paxillus* mitogenomes were calculated using DNASTAR Lasergene v7.1 (http://www.dnastar.com/). We then used the following formulas to assess strand asymmetries of the two mitogenomes: AT skew = [A - T] / [A + T], and GC skew = [G - C] / [G + C] (Wang et al. 2017). The synonymous (Ks) and nonsynonymous (Ka) substitution rates for core PCGs in the two mitogenomes were calculated with DnaSP v6.10.01(Rozas et al. 2017). MEGA v6.06 (Caspermeyer 2016) was used to calculate the overall mean genetic distances between each pair of the 15 core PCGs (*atp6, atp8, atp9, cob, cox1, cox2, cox3, nad1, nad2, nad3, nad4, nad4L, nad5, nad6,* and *rps3*), using the Kimura-2-parameter (K2P) substitution model.

### Repetitive element analysis

To identify if there were interspersed repeats or intra-genomic duplications of large fragments throughout the two mitogenomes, we conducted BLASTN searches (Chen et al. 2015) of each mitogenome against itself using an E-value of < 10^− 10^. Tandem repeats (> 10 bp in length) in the two mitogenomes were detected using the Tandem Repeats Finder (Benson 1999) with default parameters. Repeated sequences in the two mitogenomes were also searched by REPuter (Kurtz et al. 2001) to identify forward (direct), reverse, complemented, and palindromic (reverse complemented) repeats. To identify any gene fragments transferring between nuclear genomes and mitochondrial genomes of the two *Paxillus* species, we performed BLASTn searches of the two mitogenomes against their previously published nuclear genomes (JOMD00000000.1 and JMDR00000000.1).

### Comparative mitogenomic analysis and intron analysis

Up to now, only two mitogenomes from *Boletales* have been published (Li et al. 2019a). We compared the genome sizes, base compositions, gene numbers, intron numbers, and gene arrangements among different *Boletales* species to assess variations and conservativeness among the mitogenomes. Group I introns in *cox1* genes of the 61 published *Basidiomycota* mitogenomes were classified into different position classes (Pcls) according to the method of Férandon et al. (Ferandon et al. 2010). Each Pcl was constituted by introns inserted at the same position in the coding region of the *cox1* gene. The same Pcl from different species usually has a high sequence similarity, and contains orthologous intronic ORF. The Pcls of *cox1* gene were named by letters according to the similarity with the described Pcls (Ferandon et al. 2010). The conservativeness and variations of sequences around intron insertion sites (− 15 bp - 15 bp) were evaluated using WebLogo (Crooks et al. 2004).

### Phylogenetic analysis

In order to investigate the phylogenetic status of the two *Paxillus* species within the *Basidiomycota*, we constructed a phylogenetic tree of 62 species based on the combined mitochondrial gene set (15 core PCGs + 2 rRNA genes) (Li et al. 2018d). *Annulohypoxylon stygium* (*Ascomycota*) was used as outgroup (Deng et al. 2018). Individual mitochondrial genes were first aligned using MAFFT v7.037 (Katoh et al. 2017), and these alignments were then concatenated in SequenceMatrix v1.7.8 (Vaidya et al. 2011) to obtain a combined mitochondrial gene set. Potential phylogenetic conflicts between different genes were detected by a partition homogeneity test; PartitionFinder 2.1.1 (Lanfear et al. 2017) was used to determine best-fit models of evolution and partitioning schemes. Both Bayesian Inference (BI) and Maximum Likelihood (ML) methods were used to construct phylogenetic trees. MrBayes v3.2.6 (Ronquist et al. 2012) was used to perform the BI analysis, and RAxML v 8.0.0 (Stamatakis 2014) the ML analysis.

### Data availability

The complete mitogenomes of *Paxillus involutus* and *P. rubicundulus* were deposited in GenBank under accession numbers MK993563 and MK993564, respectively.

## RESULTS

### Genome features and PCGs of *Paxillus* mitogenomes

**Fig. 1:**
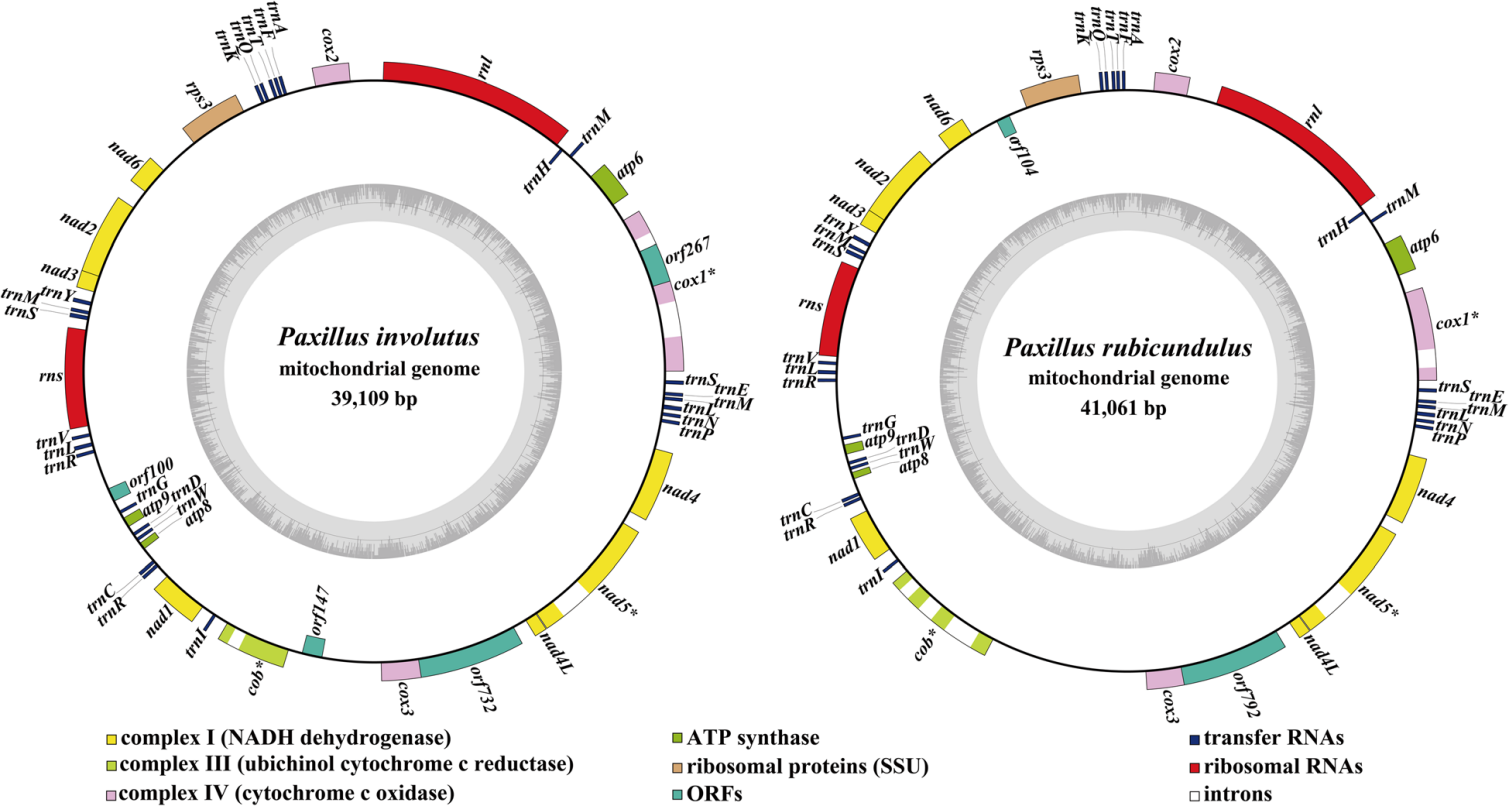
Circular maps of the mitogenomes of two *Paxillus* species. Genes are represented by different colored blocks. Coloured blocks outside each ring indicate that the genes are on the direct strand, while coloured blocks within the ring indicates that the genes are located on the reverse strand

The mitogenomes of both species were both composed of circular nucleotide molecules, of 39,109 bp and 41,061 bp, respectively (Fig. 1). The average GC content of the two mitogenomes was 21.39%. Both species contained negative AT skews and positive GC skews (Table S1).

There were 19 and 17 PCGs detected in the *P. involutus* and *P. rubicundulus* mitogenomes, respectively (Table S2), of which 78.95–82.35% were located in the direct strand. Both the mitogenomes contained 15 core PCGs, including 14 core PCGs for energy metabolism and one *rps3* gene for transcriptional regulation. In addition, the *P. involutus* mitogenome contained a PCG located in the second intron of the *cox1* gene, which encoded LAGLIDADG endonuclease. *Paxillus rubicundulus* contained a PCG encoding DNA polymerase. Three and one PCGs with unknown functions were detected in the *P. involutus* and *P. rubicundulus* mitogenomes, respectively. *P. involutus* and *P. rubicundulus* contained a homologous PCG showing no significant similarity with known protein sequences in the public databases checked; this encoded 732 amino acids in the *P. involutus* mitogenome and 792 amino acids in that of *P. rubicundulus*.

### rRNA genes and tRNA genes

Both the *Paxillus* mitogenomes contained two rRNA genes, the large subunit ribosomal RNA (*rnl*) and small subunit ribosomal RNA (*rns*) genes (Table S2). The length of the rRNA genes varied between the two: there were five nucleotide length variations between the two *rnl* genes, and nine between the two *rns* genes.

The two mitogenomes both contained 25 tRNA genes, which encode 20 standard amino acids (Table S2). Both contained two tRNAs that code for arginine and serine with different anticodons, two coding for leucine, and three coding for methionine with the same anticodons. All tRNAs in the two mitogenomes were folded into a classical cloverleaf structures (Fig. S1), with each 71–86 bp long. Three tRNA genes, including *trnY*, *trnS* and *trnL* contained large extra arms, which resulted in their tRNA gene length exceeding 85 bp. Of the 25 tRNA genes shared by the two *Paxillus* mitogenomes, 10 contained sites that varied between the two. Thirteen variable sites were detected in the 25 tRNAs between the two species, of which 53.85% occurred on the acceptor arm, suggesting that the acceptor arm was highly variable in the two *Paxillus* mitogenomes.

### Intergenic sequence and mitogenome composition

Both the mitogenomes contained two overlapping regions (Table S2), one of which was located between the *nad2* and *nad3* genes (13 bp). Another overlapping region in *P. involutus* was located between *cox3* and orf732 (46 bp), and a further one in *P. rubicundulus* was across the neighboring *cox3* and orf792 genes (88 bp). The two *Paxillus* mitogenomes lacked the common overlapping region found in some other *Basidiomycota* mitogenomes located between the *nad4L* and *nad5* genes. A total of 11,295 bp and 13,790 bp intergenic sequences were detected in the *P. involutus* and *P. rubicundulus* mitogenomes, respectively, with each intergenic sequence ranging from 9 bp to 3537 bp. The longest intergenic sequence detected was between *cob* and *cox3* in the *P. rubicundulus* mitogenome.

**Fig. 2:**
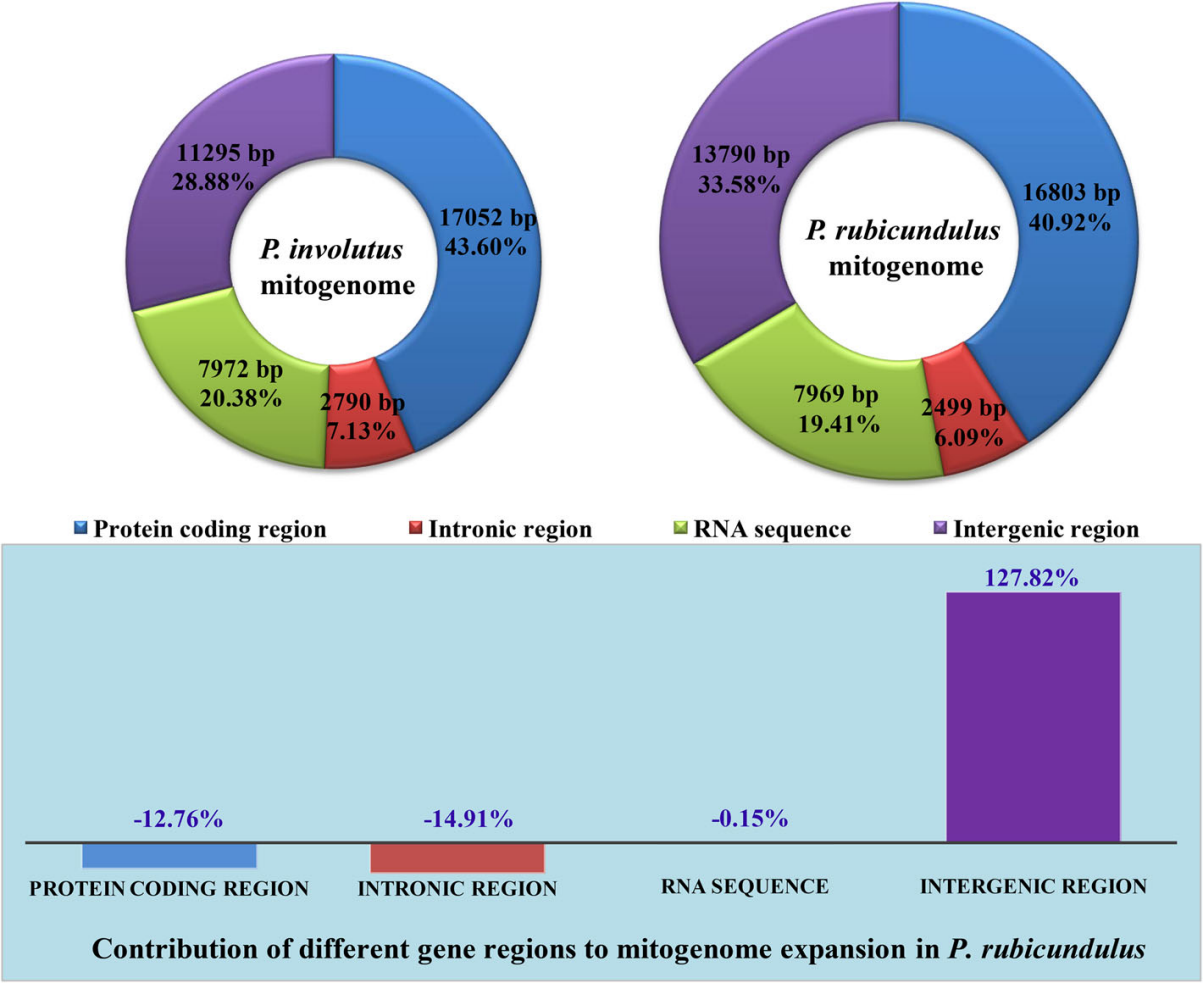
The protein-coding, intronic, intergenic, and RNA gene region proportions of the entire mitogenomes of the two *Paxillus* species. The bottom panel shows the contribution of different gene regions to the expansion of the *P. rubicundulus* mitogenome

Protein coding regions accounted for the largest proportion (40.92–43.60%) of the two *Paxillus* mitogenomes, followed by the intergenic region, which accounted for 20.88–33.58% of the whole mitogenomes (Fig. 2). RNA regions, including rRNA and tRNA, accounted for 19.41–20.38% of the two mitogenomes. The intronic region accounted for the smallest proportion of the two mitogenomes, with only 6.09–7.13%. Compared with the *P. involutus* mitogenome, the size of that of *P. rubicundulus* was expanded by 1952 bp, to which the intergenic region contributed 127.82% of the size expansion, while the other regions contracted in the *P. rubicundulus* mitogenome relative to rgose in that of *P. involutus*, indicating that the intergenic region was the most important factor contributing to the dynamic changes of mitogenomes in the two *Paxillus* species.

### Repetitive elements in the two *Paxillus* mitogenomes

A total of 53 and 47 tandem repeats were detected in the *P. involutus* and *P. rubicundulus* mitogenomes, respectively (Table S3). The longest tandem repeat sequence was located in the *P. rubicundulus* mitogenome, with a length of 49 bp. Most of the tandem repeats contained 2–4 copies in the two mitogenomes. Using REPuter, we detected 1 complemented, 24 forward, 4 palindromic, and 21 reverse repeats in the *P. involutus* mitogenome, accounting for 3.13% of the whole mitogenome (Table S4). Repeated sequences identified by REPuter accounted for 3.25% of the *P. rubicundulus* mitogenome.

In order to detect whether there were gene fragments that naturally transferred between the mitochondrial and nuclear genomes of the two *Paxillus* species, we compared the two newly sequenced mitogenomes with their published nuclear genomes (Kohler et al. 2015) by BLASTN. Eleven repetitive fragments were identified between the mitochondrial and nuclear genomes of *P. involutus*, ranging from 33 bp to 3202 bp in length (Table S5). The pair-wise nucleotide similarities of these repetitive fragments ranged from 85.31–100%. The repetitive sequences detected in the *P. involutus* mitogenome accounted for 10.64% of the entire mitogenome. A total of 62 repetitive gene fragments were detected between the *P. rubicundulus* mitochondrial and nuclear genomes, with pair-wise nucleotide similarities ranging from 87.93–100%. The largest repeat fragment in the *P. rubicundulus* mitogenome reached 2367 bp, which contained a partial sequence of the *rnl* gene, the whole sequence of the *cox2* gene, and the intergenic sequences around them. The repetitive gene fragments between *P. rubicundulus* mitochondrial and nuclear genomes accounted for 36.50% of the entire mitogenome.

### Mitochondrial gene arrangement in *Basidiomycota* species

**Fig. 3:**
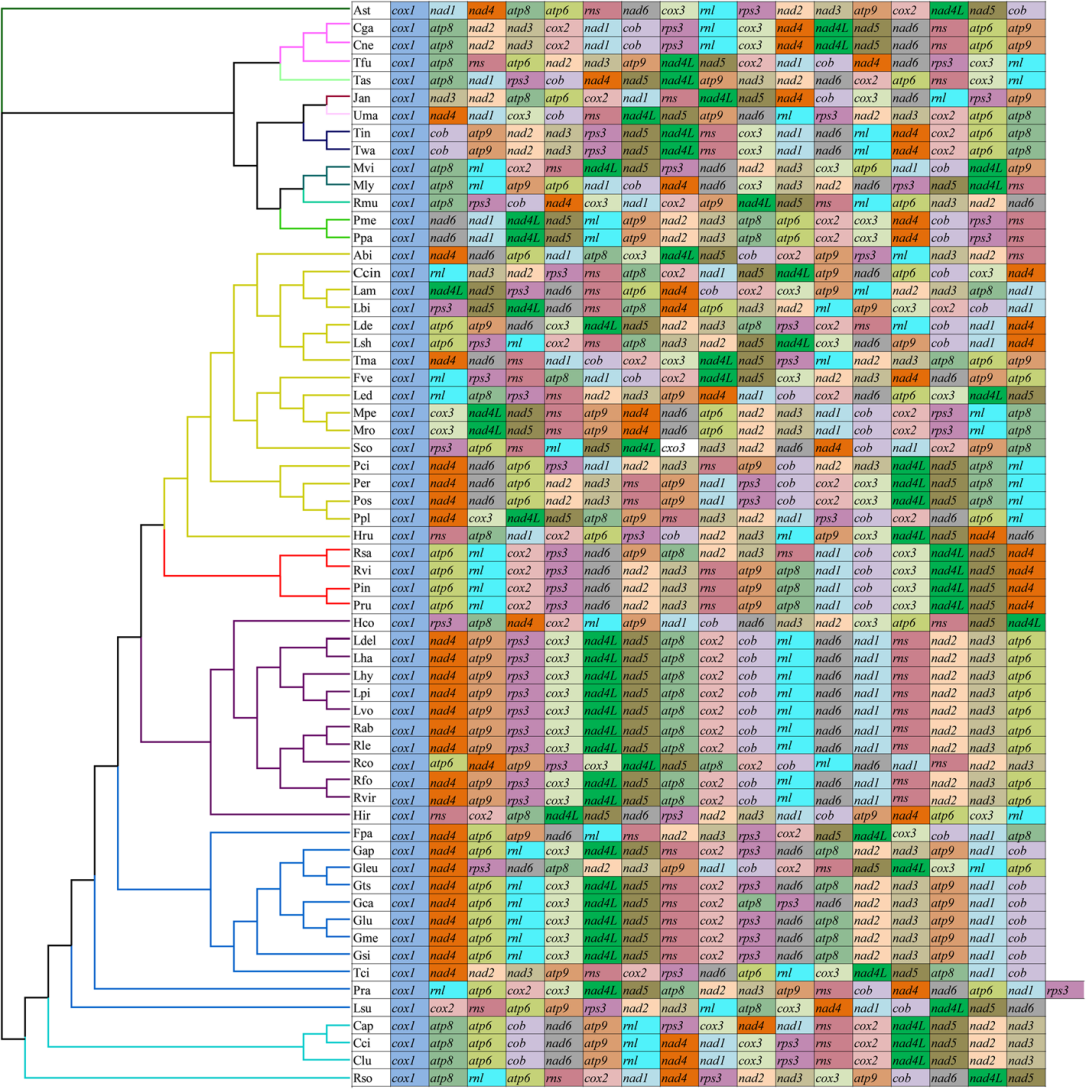
Mitochondrial gene arrangement analyses of 61 species from *Basidiomycota*. Genes are represented with different colour blocks. All genes are shown in order of occurrence in the mitochondrial genome, starting from *cox1*. Fourteen core protein coding genes, one *rps3* gene, and two rRNA genes were included in the gene arrangement analysis. Species and NCBI accession number used for gene arrangement analysis in the present study are listed in Supplementary Table S6

The mitochondrial gene arrangement in *Basidiomycota* varied greatly at the order level (Fig. 3). Of the 13 orders in *Basidiomycota* with data, no identical arrangement of mitochondrial genes was found between any two orders, suggesting that large-scale rearrangements of *Basidiomycota* mitochondrial genes had occurred in the course of evolution. At the level of family, only some species from *Paxillaceae* and *Rhizopogonaceae* were found to have the same gene order. Gene orders in other families were highly variable. At the generic level, we found large-scale gene rearrangement events in aevearl genera, including *Cantharellus, Ganoderma, Lyophyllum, Pleurotus,* and *Russula*. However, the gene arrangement of the two species of *Paxillus* studied was found to be conservative.

### Variation, genetic distance, and evolutionary rates of core PCGs

**Fig. 4:**
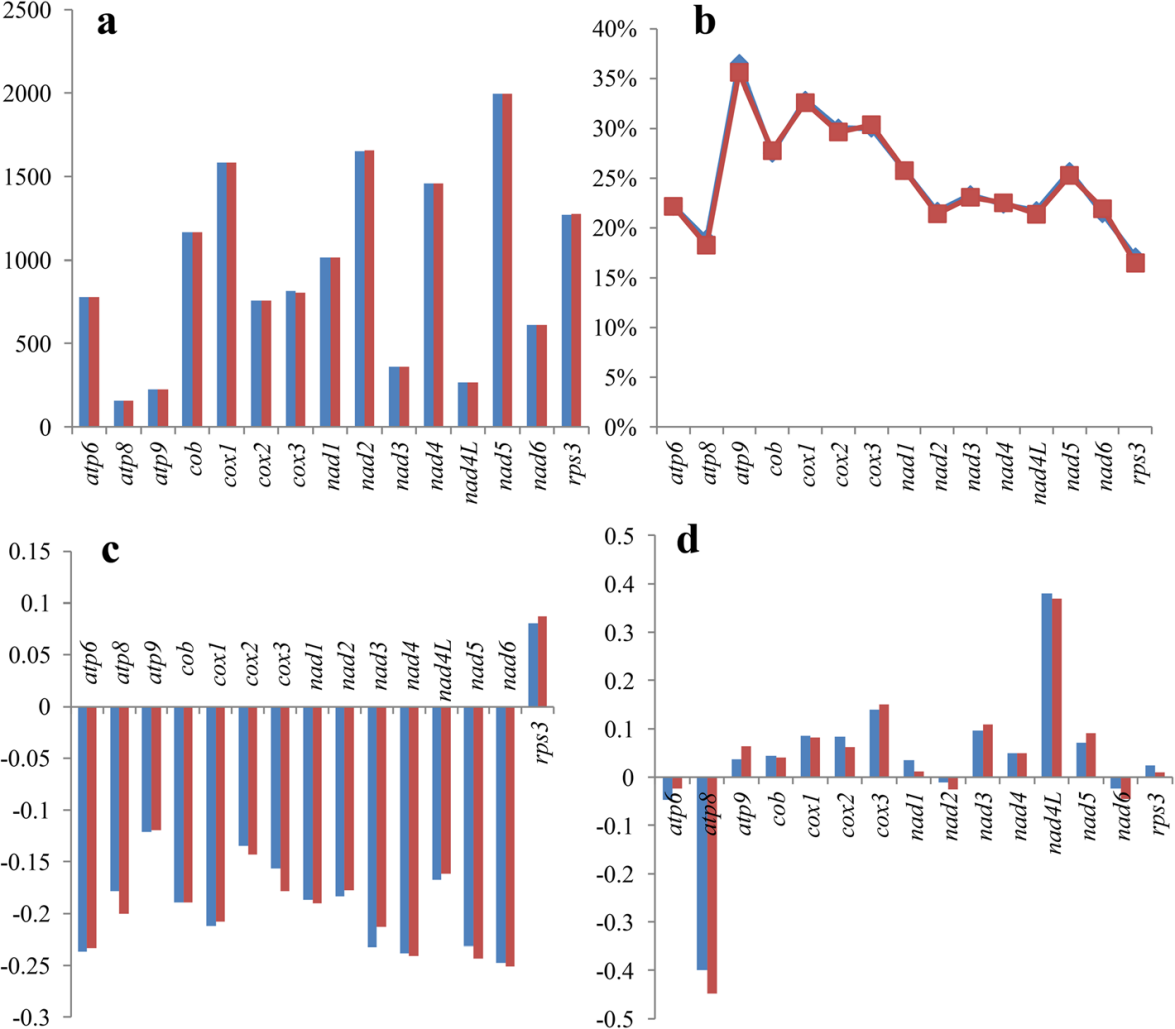
Variation in the length and base composition of each of 15 protein-coding genes (PCGs) between two *Paxillus* mitogenomes. **a** PCG length variation; **b** GC content of the PCGs; **c** AT skew; **d** GC skew

Among the 15 core PCGs detected, three genes showed length variations between the two *Paxillus* species, including *cox3*, *nad2* and *rps3* (Fig. 4). Among them, *cox3* and *nad2* genes showed two amino acid length variations between the two, while *rps3* had one amino acid length variation between them. The GC content of 13 of the 15 core PCGs differed between the two *Paxillus* species, except for that of the *atp6* and *nad1* genes. Among the 15 core PCGs tested, *atp9* had the highest GC content and *atp8* the lowest. Most AT skews of core PCGs were negative, except for the *rps3* gene. Eleven of the 15 core PCGs showed positive GC skews, while *atp6*, *atp8*, *nad2* and *nad6* had negative GC skews.

**Fig. 5:**
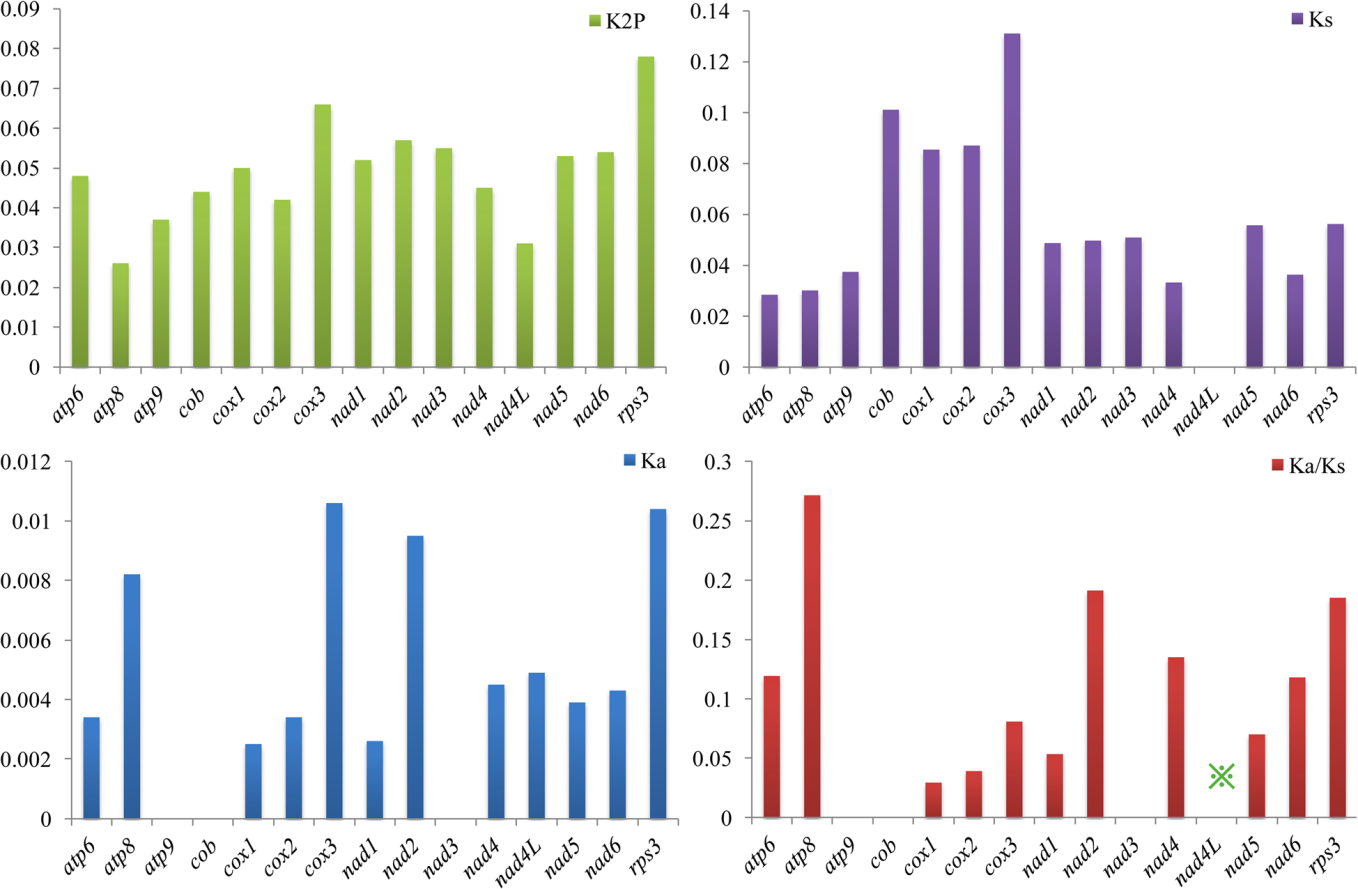
Genetic analysis of 15 protein coding genes conserved in two *Paxillus* mitogenomes. K2P, the Kimura-2-parameter distance; Ka, the mean number of nonsynonymous substitutions per nonsynonymous site; Ks, the mean number of synonymous substitutions per synonymous site. The asterisk indicates that the gene is under strong pressure of positive selection

Among the 15 core PCGs detected, *rps3* contained the largest K2P genetic distance between the two species (Fig. 5), followed by *cox3* and *nad2*, which suggested that these genes were largely differentiated in the *Paxillus* species. The K2P genetic distance of *atp8* was the lowest among the 15 core PCGs between the two mitogenomes, indicating that this gene was highly conserved in the two species. The *cox3* gene exhibited the highest synonymous substitution (Ks) rate, while *nad4L* had the lowest substitution rate among the 15 core PCGs detected. The highest non-synonymous substitution rate (Ka) was in *cox3*, while *atp9*, *cob* and *nad3* exhibited the lowest Ka values among the 15 core PCGs. The Ka/Ks values for most core PCGs were less than 1, indicating that these genes were subjected to purifying selection. However, the Ka/Ks value of *nad4L* gene was far greater than 1, indicating that this gene has been subjected to strong positive selection in the two *Paxillus* species. A non-synonymous mutation was detected in the *nad4L* gene, of which the 46th nucleotide ‘G’ in *P. involutus* mutated into nucleotide ‘A’ in *P. rubicundulus*.

### Intron dynamics of *cox1* gene in *Basidiomycota*

**Fig. 6:**
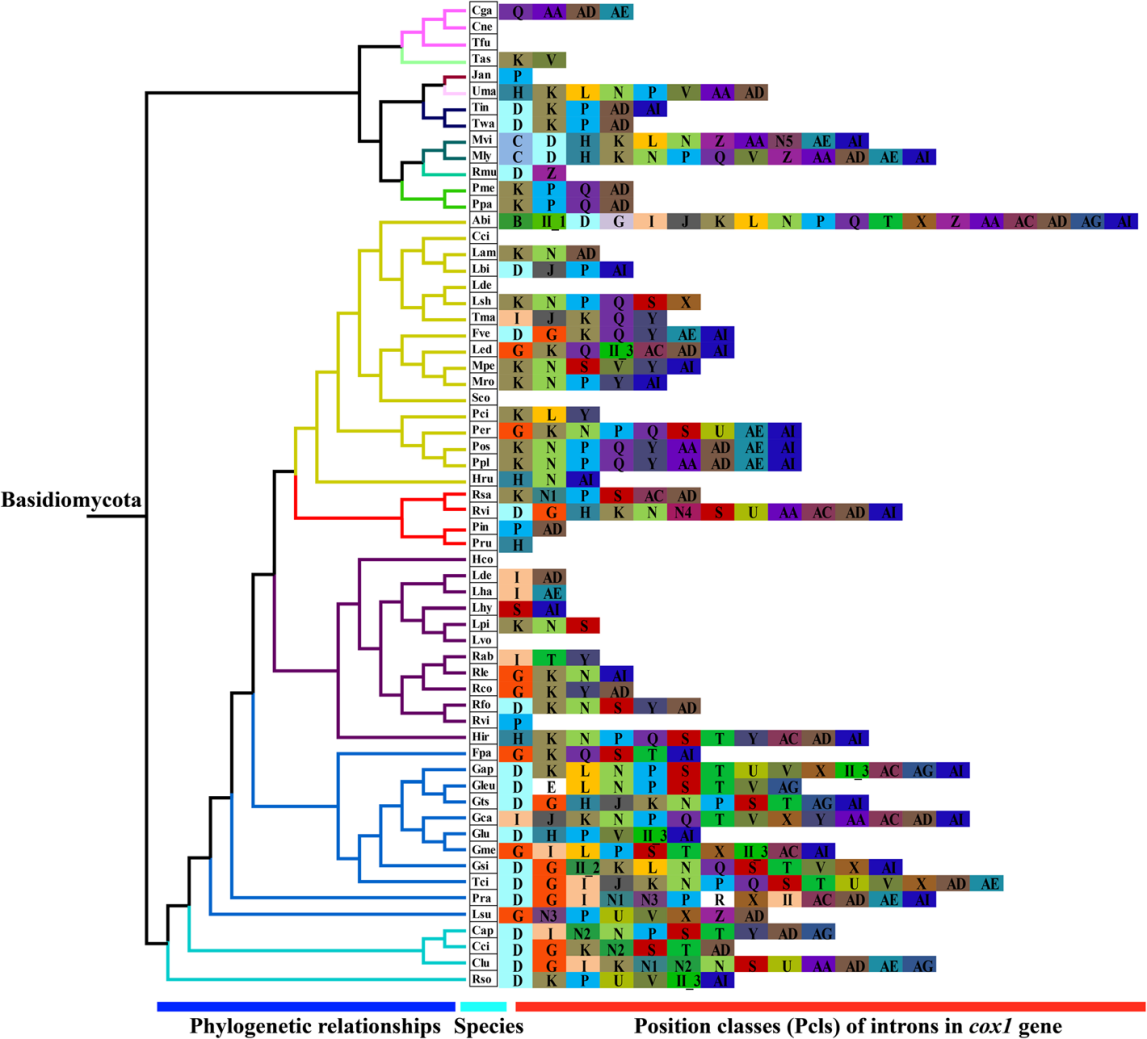
Position class (Pcl) information of *cox1* genes of 61 *Basidiomycota* species. The same Pcl (orthologous intron) is represented by the same letter. The phylogenetic positions of the species were established using the Bayesian inference (BI) method and Maximum Likelihood (ML) method based on 15 concatenated mitochondrial core proteins and 2 rRNA genes. N1 - N5 represent the newly detected Pcl in this study; The II in the figure shows the intron belongs to the group II intron. Species ID are shown in Supplementary Table S6

According to previous studies (Ferandon et al. 2013), introns can be divided into different Pcls according to their precise insertion sites in the protein coding region of PCGs. Introns belonging to the same Pcls were considered as homologous and often contained homologous intronic open reading frames (ORFs). Introns belonging to different Pcls were considered as non-homologous introns, and showed low sequence similarities. In the present study, 367 introns were detected in *cox1* genes of 61 *Basidiomycota* species, including 359 group I introns and 8 group II introns. The number of introns in the *cox1* gene of individual species ranged from 0 to 19. A total of 32 position classes (Pcls) of group I introns were detected in the 61 species (Fig. 6), including five novel Pcls that had not been identified in previous studies, and 27 known Pcls. Pcls K was the most common intron class, which occurred in 36 of the species. Pcls P, AD, AI, N, and S were present in more than 30% of the species tested, and considered as the common Pcls in *Basidiomycota*. Pcls E was detected only in the *cox1* gene of *Ganoderma leucocontextum* (MH252534) (Li et al. 2019e). However, it was present in the *cox1* gene of the distantly related aquatic species *Blastocladiella emersonii* (Tambor et al. 2008) which belongs to the phylum *Blastocladiomycota*, a situation not readily explained as horizontal gene transfer between fungi which grow in such different environments seems improbable. In the two *Paxillus* species, we detected three group I introns, which belonged to different Pcls, indicating great variation of introns between them.

**Fig. 7:**
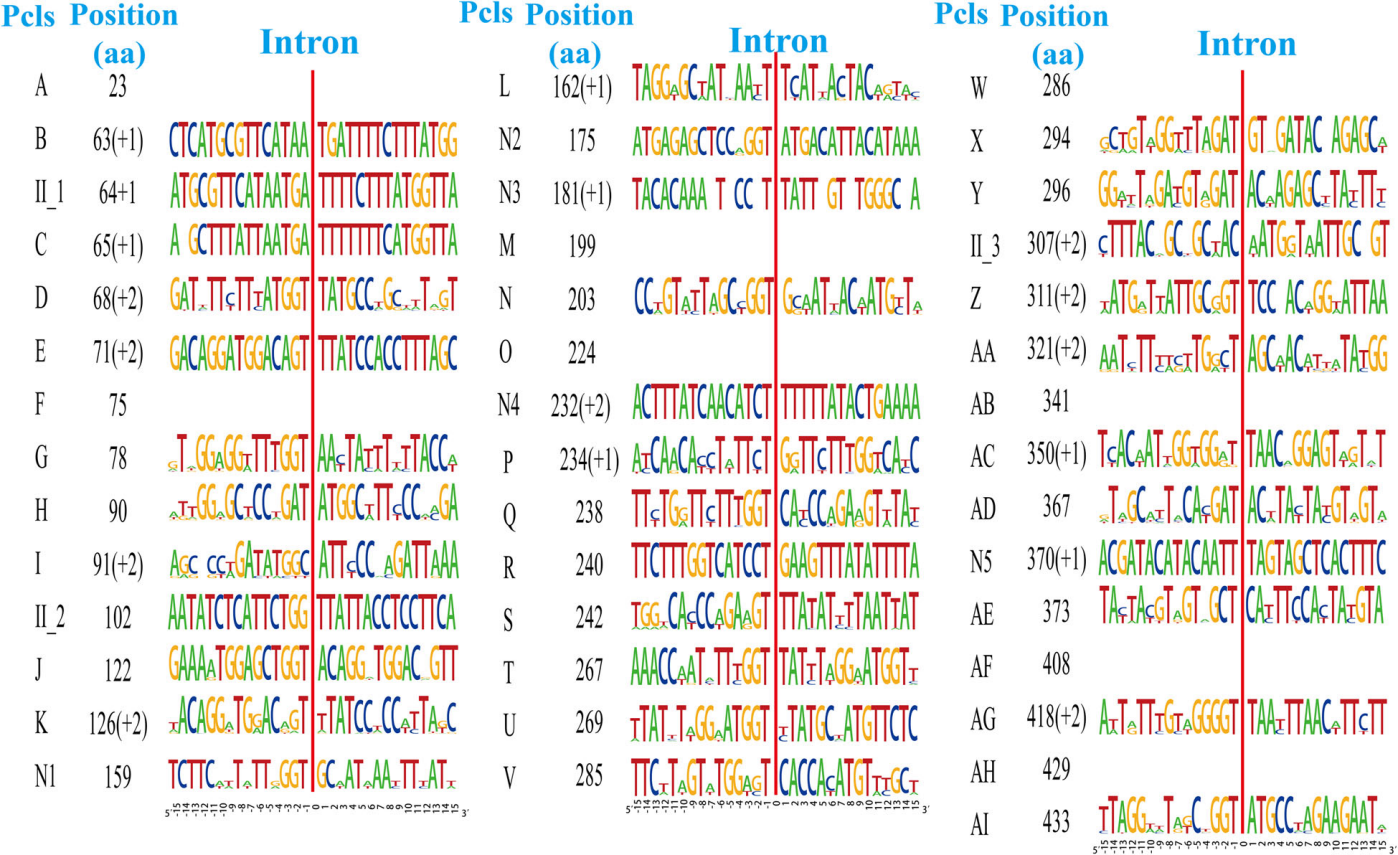
The insertion sites of different Pcls in the coding region of 61 Basidiomycete cox1 genes and the conservative analysis of the sequence around the insertion sites (− 15 bp – 15 bp). The symbols “+ 1” and “+ 2” indicate that the insertion of the intron occurs inside the indicated codon: between the nt 1 and nt 2 of this codon for “+ 1” and between the nt 2 and nt 3 for “+ 2”

The conservativeness and variation of nucleic acid sequences (30 bp) around insertion sites of different Pcls was also assessed (Fig. 7). We found that the insertion site preferences of different Pcls were quite different, while the nucleic acid sequences around the same Pcls were relatively conservative. Over 13 of the 30 nucleic acid loci detected were highly consistent around the same Pcls. Of the 32 group I intron Pcls we detected, 28 were inserted downstream of base T, while 11 Pcls were inserted behind GGT. In addition, 14 Pcls of 32 group I introns were located before base A, indicating a preference of intron insertion sites. These results could be useful for accurately identifying intron classes and annotating mitochondrial genes with multiple introns.

### Comparative mitogenome analysis and phylogenetic analysis

Compared with previously published *Rhizopogon* mitogenomes, the two *Paxillus* species contained smaller mitogenomes (Table S1). That of *P. involutus* was 97.16% smaller than the largest mitogenome, *R. vinicolor*. The two *Paxillus* species contained a higher proportion of protein coding regions, RNA regions, and intergenic regions, but a lower proportion of intronic regions relative to the *Rhizopogon* species. Amongst the four *Boletales* species we tested, they all contained negative AT skews and positive GC skews. The two *Paxillus* species contained less PCGs, intron and intronic ORFs than *Rhizopogon* species. However, the four species from *Boletales* had the same number of rRNA genes and tRNA genes.

**Fig. 8:**
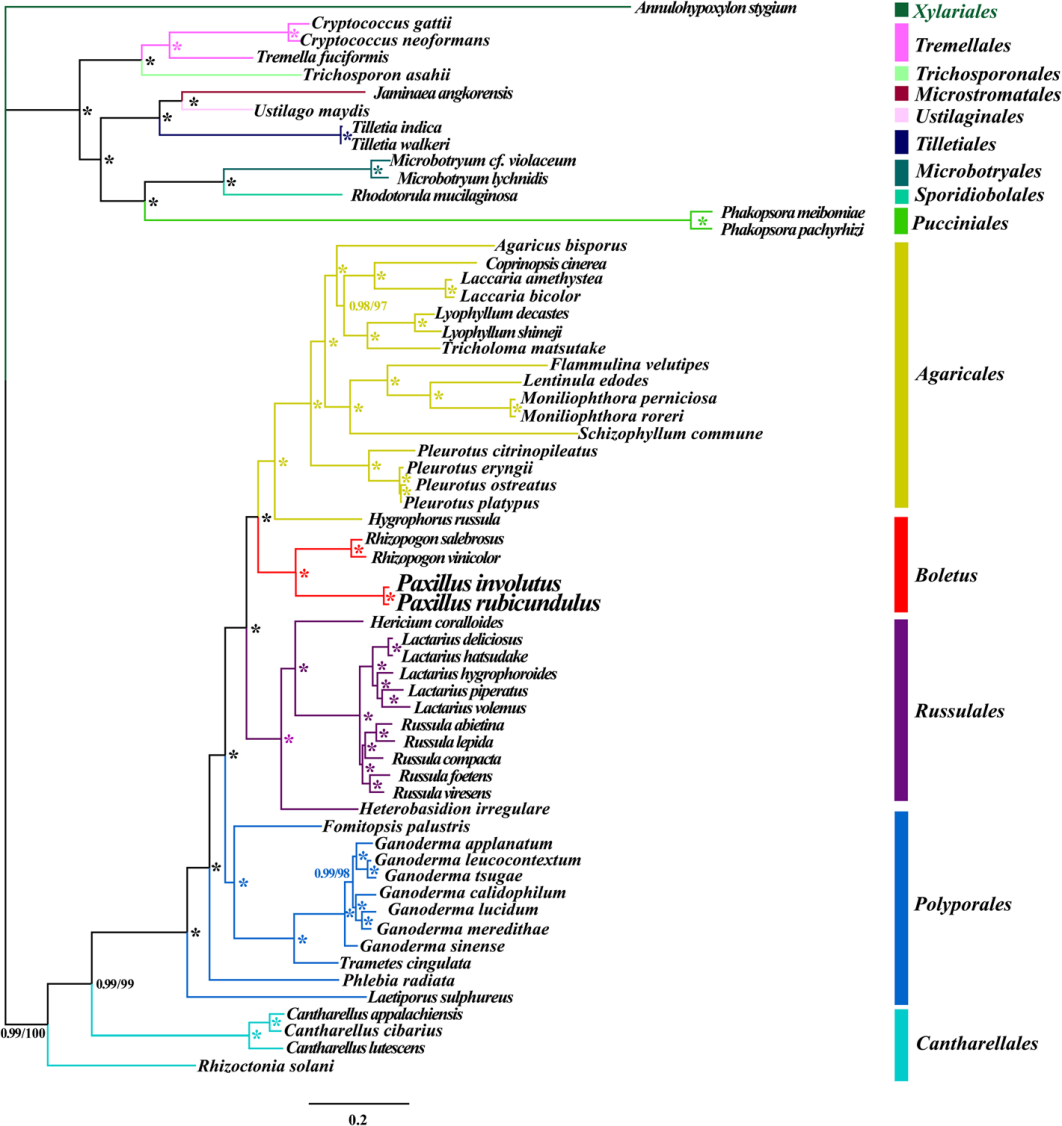
Molecular phylogeny of 61 *Basidiomycete* species based on Bayesian inference (BI) and Maximum likelihood (ML) analysis of 15 protein coding genes and two rRNA genes. Support values are Bayesian posterior probabilities (before slash) and bootstrap (BS) values (after slash). The asterisk indicates that the BPP value is 1 and the BS value is 100 of the branch. Species and NCBI accession numbers for genomes used in the phylogenetic analysis are provided in Supplementary Table S6

Phylogenetic analysis using Maximum likelihood (ML) and Bayesian Inference (BI) methods based on the combined mitochondrial gene set (14 core PCGs + *rps3* + 2 rRNA genes) yielded identical and well-supported tree topologies (Fig. 8). All major clades within the trees were well supported (BS ≥ 97; BPP ≥0.98). According to the phylogenetic tree, the 61 *Basidiomycota* species included could be divided into 13 major clades, corresponding to the orders *Agaricales*, *Boletales*, *Cantharellales*, *Microbotryales*, *Microstromatales*, *Polyporales, Pucciniales, Russulales*, *Sporidiobolales*, *Tilletiales*, *Tremellales*, *Trichosporonales*, and *Ustilaginales* (Table S6). *Boletales* had a close phylogenetic relationship with *Agaricales* and *Russulales,* and the four *Boletales* species could be divided into two groups, with *P. involutus* as a sister species to *P. rubicundulus*.

## DISCUSSION

### Genome size variation in *Boletales*

The mitochondrial genome size of fungi is highly variable, the largest being in *Rhizoctonia solani* (235.85 kb; (Losada et al. 2014) and the smallest in *Spizellomyces punctatus* (1.14 kb; (Forget et al. 2002). Intronic and intergenic regions, the content of repeat sequence and horizontally transferred genes have been found to be the main factors contributing to size dynamic changes in fungal mitogenomes (Losada et al. 2014; Salavirta et al. 2014). So far, only four complete mitogenomes from *Boletales* have been published, and these also varied in size. The two *Rhizopogon* species contained larger mitogenomes than those from *Paxillus*, was mainly due to their larger intronic regions. In *Paxillus*, the intergenic region was the main factor contributing to the size variation of the mitogenomes. Intronic and intergenic regions together contribute most to variations in mitogenome size in *Boletales*, as well as in other *Basidiomycota* so far examined (Ferandon et al. 2010).

### Gene content variation in *Paxillus* species

Since eukaryotic organisms first acquired a mitogenome from endosymbiotic bacteria, most mitochondrial genes have been transferred to the nuclear genome during evolution, which is considered to have many advantages (Adams and Palmer 2003). However, most fungal mitochondria have so far been found to retain 14 core PCGs for energy metabolism and one *rps3* gene for transcriptional regulation (Allen 2015; Bjorkholm et al. 2015). We found that the core PCGs in the two *Paxillus* species varied in base composition and length. In addition, the two core PCGs had different genetic distances between the species, indicating that different genes evolve at different rates. The *nad4L* gene is an important functional protein for ATP synthesis in mitochondria, which plays an important role in maintaining cell homeostasis and regulating celluar responses to the environment (Kazama et al. 2001). Interestingly, we found that the *nad4L* gene had experienced a strong positive selection between the two *Paxillus* species, which might be related to their host specificity. In addition, several PCGs coding homing endonucleases, DNA polymerase, and several unknown ORFs, were found in the species, indicating that some PCGs remain to be revealed. The tRNA and rRNA genes shared by the two *Paxillus* species also underwent base and length variations. Base variation in tRNA has been thought to affect the efficiency of amino acid transport and protein synthesis in animals (Ding et al. 2019; Lin et al. 2019). Further studies are needed to reveal the effect of tRNA variation on the growth and development of basidiomycete species.

### Potential gene transfer between nuclear genome and mitogenome

The synergistic effects of nuclear and mitochondrial genes maintain the growth and development of eukaryotes (Baris et al. 2017; Latorre-Pellicer et al. 2016). During the evolution of eukaryotes, most mitochondrial genes have been transferred to the nucleus (Adams and Palmer 2003). However, some nuclear genes have also been found transferred to the mitogenome, and this has affected the evolution of eukaryotes (Li et al. 2019f; Srirattana and St John 2018). In our study, 10.64 and 36.50% of gene fragments were found potentially transferred between nuclear and mitochondrial genomes of the two *Paxillus*, respectively, which is a higher proportion than reported from *Ganoderma lucidum* (Li et al. 2013) and *Pleurotus eryngii* (Li et al. 2018a). Our result indicates that frequent natural gene transfer has occurred in the two *Paxillus* species. The impact of this transfer on the evolution and function of the *Paxillus* mitogenomes, however, remains unknown.

### Gene rearrangement of basidiomycete species

The evolutionary rate of mitochondrial gene in fungi has been reported as intermediate between that of animals and plants (Aguileta et al. 2014; Barr et al. 2005). As more mitochondrial genomes have been sequenced, gene rearrangements found to be widespread in animals, plants, and fungi (Park et al. 2016; Sankoff et al. 1992). Various gene rearrangement models have been proposed to reveal gene rearrangement events in animals (Lavrov et al. 2002; Xia et al. 2016), but mitochondrial arrangements in fungi have been much less studied. We found that the mitochondrial gene arrangement in *Basidiomycota* was highly variable. At the order level, no two orders had the same gene order. In addition, we also observed large-scale gene rearrangements at the family or even generic level (Li et al. 2018a; Li et al. 2018b; Li et al. 2019b; Li et al. 2019c). The *Basidiomycota* mitogenome contains more repetitive sequences than animals, which we consider to be one of the main factors resulting in the high variation seen in the mitochondrial genome; this result is consistent with the findings of previous studies (Aguileta et al. 2014).

### Dynamic changes of introns in *cox1* gene of *Basidiomycota*

As a mobile genetic element (Burke 1988; Repar and Warnecke 2017), introns have been considered one of the main factors contributing to the variations of mitogenome organization and size (Hamari et al. 2002; Kanzi et al. 2016; Repar and Warnecke 2017). In *Basidiomycota*, the *cox1* gene was found to harbour the most introns, with the largest proportion belonging to group I (Li et al. 2020). We detected 32 Pcls of the group I introns and 3 Pcls of the group II introns amongst the 61 species for which data were available. Among these intron Pcls, five were newly identified here, indicating that the phylum contains diverse intron types. Pcl K was the most widely distributed Pcl in *Basidiomycota*, and so was presumed to be derived from the phylum’s ancestors. Pcl E was only detected in *Ganoderma leucocontextum* (Li et al. 2019e)*,* but this has also been reported in the distantly related *Blastocladiella emersonii* (Tambor et al. 2008), something suggestive of horizontal gene transfer, but difficult to imagine in view of the different ecologies of the two phyla. Different *Basidiomycota* species, even from within the same genera, could contain different numbers and Pcls of introns, indicating frequent intron loss or acquisition events during the evolution of the phylum. Introns contained homing endonucleases that can insert into specific sites in the mitogenome could potentially be used as gene editing tools (Chi et al. 2018; Zubaer et al. 2018). We assessed the conservativeness and variability of nucleic acid sequences around insertion sites of introns from basidiomycetes where information was available which showed that the insertion sites had certain base preferences. Our results provide a reference source for the accurate annotation of basidiomycete mitogenomes and also indicate a potential application of homing endonucleases in gene editing.

### Phylogenetic relationships of *Basidiomycota* based on mitochondrial genes

Because of their independence from the nuclear genome, their rapid evolutionary rate, and the presence of many molecular markers, the mitogenome has become a powerful tool for studying phylogeny, population genetics, and taxonomy (Cameron 2014; Li et al. 2015; Li et al. 2019d). With the rapid development of next generation sequencing technology, more and more mitogenomes have been acquired, which has promoted the development of eukaryotic systems biology (Johri et al. 2019; Wang et al. 2018). However, the mitogenomes of fungi have been less studied than those of animals. Up to now, few mitochondrial molecular markers have been employed in studying their population genetics or phylogeny (Li et al. 2020). We obtained identical and well-supported tree topologies by BI and ML revealing phylogenetic relationships amongst 61species of *Basidiomycota*, based on the combined mitochondrial gene set. This demonstrates that the mitochondrial gene is a reliable molecular marker for the analysis of phylogenetic relationships of basidiomycetes and perhaps other fungal groups More mitogenomes are, however, needed to comprehensively assess the origin and evolution of the *Basidiomycota*.

## CONCLUSION

In this study, the mitogenomes of two *Paxillus* species were assembled, annotated, and analyzed. Comparative mitogenome analysis indicated that the intronic and intergenic regions were the main factors contributing to mitogenome size variations in *Boletales*. The genome content, gene length, base composition, tRNAs, and rRNAs also varied greatly between the two *Paxillus* species studied. In addition, the *nad4L* gene was found to have been subjected to strong positive selection in the two species, which we suggest may be related to their contrasting ecological strategies. Potential gene fragment transferance between mitochondrial and nuclear genomes was also detected in the two *Paxillus* species.

Large-scale gene rearrangements were also found to have occurred in basidiomycete species for which data were avaliable, with introns experiencing frequent loss or gain events, resulting in large variations of mitogenome organization and size in *Basidiomycota*. In addition, the insertion sites of introns in basidiomycete mitogenomes were found to have a certain base preference. Phylogenetic analysis revealed that mitochondrial genes were reliable tools for analyzing phylogenetic relationships within *Basidiomycota*. As the first report on the mitogenome from the genus *Paxillus* or even the suborder *Paxilineae* of *Boletales*, this study contributes to our understanding of the evolution, genetics and phylogeny of both *Paxillus* species and the phylum *Basiiomycota* as a whole.

## Supplementary information

**Additional file 1 Fig. S1.** Putative secondary structures of the 25 tRNA genes identified in the mitogenomes of two *Paxillus* species. Residues conserved across the two mitogenomes are shown in green, while variable sites are shown in red. All genes are shown in order of occurrence in the mitogenome of *P. involutus*, starting from *trnM*.

**Additional file 2 Table S1.** Comparison on mitogenomes between four *Boletales* species. **Table S2.** Gene features and organization of the *Paxillus* mitogenomes. **Table S3.** Tandem repeats detected in the mitogenomes of two *Paxillus* species using the Tandem Repeats Finder. **Table S4.** Distribution of repeat loci in the two *Paxillus* mitogenomes searched by REPuter. **Table S5.** Local BLAST searches between the mitochondrial and the nuclear genomes of two *Paxillus* species. **Table S6.** Species and GenBank accession number used for phylogenetic analysis in this study.

## Data Availability

All data generated or analyzed during this study are included in this published article [and its supplementary information files].

## Abbreviations

mitogenome: Mitochondrial genome
BI: Bayesian inference
ML: Maximum likelihood
ECMF: Ectomycorrhizal fungi
PCG: Protein-coding gene
PCWDEs: Plant cell wall–degrading enzymes
Pcls: Position classes
Ks: Synonymous substitution rates
Ka: Nonsynonymous substitution rates

## References

[CR1] Adams KL, Palmer JD (2003). Evolution of mitochondrial gene content: gene loss and transfer to the nucleus. Molecular Phylogenetics and Evolution.

[CR2] Aguileta G, de Vienne DM, Ross ON, Hood ME, Giraud T, Petit E, Gabaldon T (2014). High variability of mitochondrial gene order among fungi. Genome Biology and Evolution.

[CR3] Allen JF (2015). Why chloroplasts and mitochondria retain their own genomes and genetic systems: colocation for redox regulation of gene expression. Proceedings of the National Academy of Sciences of the United States of America.

[CR4] Andersen MM, Balding DJ (2018). How many individuals share a mitochondrial genome?. PLoS Genetics.

[CR5] Bankevich A, Nurk S, Antipov D, Gurevich AA, Dvorkin M, Kulikov AS, Lesin VM, Nikolenko SI, Pham S, Prjibelski AD, Pyshkin AV, Sirotkin AV, Vyahhi N, Tesler G, Alekseyev MA, Pevzner PA (2012). SPAdes: a new genome assembly algorithm and its applications to single-cell sequencing. Journal of Computational Biology.

[CR6] Baris TZ, Wagner DN, Dayan DI, Du X, Blier PU, Pichaud N, Oleksiak MF, Crawford DL (2017). Evolved genetic and phenotypic differences due to mitochondrial-nuclear interactions. PLoS Genetics.

[CR7] Barr CM, Neiman M, Taylor DR (2005). Inheritance and recombination of mitochondrial genomes in plants, fungi and animals. The New Phytologist.

[CR8] Benson G (1999). Tandem repeats finder: a program to analyze DNA sequences. Nucleic Acids Research.

[CR9] Bernt M, Donath A, Juhling F, Externbrink F, Florentz C, Fritzsch G, Putz J, Middendorf M, Stadler PF (2013). MITOS: improved de novo metazoan mitochondrial genome annotation. Molecular Phylogenetics and Evolution.

[CR10] Bjorkholm P, Harish A, Hagstrom E, Ernst AM, Andersson SG (2015). Mitochondrial genomes are retained by selective constraints on protein targeting. Proceedings of the National Academy of Sciences of the United States of America.

[CR11] Bleasby AJ, Wootton JC (1990). Construction of validated, non-redundant composite protein sequence databases. Protein Engineering.

[CR12] Burke JM (1988). Molecular genetics of group I introns: RNA structures and protein factors required for splicing--a review. Gene.

[CR13] Cameron SL (2014). Insect mitochondrial genomics: implications for evolution and phylogeny. Annual Review of Entomology.

[CR14] Caspermeyer J (2016). MEGA evolutionary software re-engineered to handle Today's big data demands. Molecular Biology and Evolution.

[CR15] Chen C, Khaleel SS, Huang H, Wu CH (2014). Software for pre-processing Illumina next-generation sequencing short read sequences. Source Code for Biology and Medicine.

[CR16] Chen Y, Ye W, Zhang Y, Xu Y (2015). High speed BLASTN: an accelerated MegaBLAST search tool. Nucleic Acids Research.

[CR17] Chi SI, Urbarova I, Johansen SD (2018). Expression of homing endonuclease gene and insertion-like element in sea anemone mitochondrial genomes: lesson learned from Anemonia viridis. Gene.

[CR18] Coordinators NR (2017) Database resources of the national center for biotechnology information. Nucleic Acids Research 46(D1): D8-D13.10.1093/nar/gkx1095 10.1093/nar/gkx1095 PMC5753372 29140470

[CR19] Crooks GE, Hon G, Chandonia JM, Brenner SE (2004). WebLogo: a sequence logo generator. Genome Research.

[CR20] Delsuc F, Stanhope MJ, Douzery EJ (2003). Molecular systematics of armadillos (Xenarthra, Dasypodidae): contribution of maximum likelihood and Bayesian analyses of mitochondrial and nuclear genes. Molecular Phylogenetics and Evolution.

[CR21] Deng Y, Hsiang T, Li S, Lin L, Wang Q, Chen Q, Xie B, Ming R (2018). Comparison of the mitochondrial genome sequences of six Annulohypoxylon stygium isolates suggests short fragment insertions as a potential factor leading to larger genomic size. Frontiers in Microbiology.

[CR22] Ding Y, Teng YS, Zhuo GC, Xia BH, Leng JH (2019) The mitochondrial tRNAHis G12192A mutation may modulate the clinical expression of deafness-associated tRNAThr G15927A mutation in a Chinese pedigree. Current Molecular Medicine 19(2):136-146. 10.2174/1566524019666190308121552 10.2174/1566524019666190308121552 30854964

[CR23] Ferandon C, Moukha S, Callac P, Benedetto JP, Castroviejo M, Barroso G (2010). The Agaricus bisporus cox1 gene: the longest mitochondrial gene and the largest reservoir of mitochondrial group i introns. PLoS One.

[CR24] Ferandon C, Xu J, Barroso G (2013). The 135 kbp mitochondrial genome of Agaricus bisporus is the largest known eukaryotic reservoir of group I introns and plasmid-related sequences. Fungal genetics and biology : FG & B.

[CR25] Forget L, Ustinova J, Wang Z, Huss VA, Lang BF (2002). Hyaloraphidium curvatum: a linear mitochondrial genome, tRNA editing, and an evolutionary link to lower fungi. Molecular Biology and Evolution.

[CR26] Franco AR, Sousa NR, Ramos MA, Oliveira RS, Castro PM (2014). Diversity and persistence of ectomycorrhizal fungi and their effect on nursery-inoculated Pinus pinaster in a post-fire plantation in northern Portugal. Microbial Ecology.

[CR27] Hahn C, Bachmann L, Chevreux B (2013). Reconstructing mitochondrial genomes directly from genomic next-generation sequencing reads--a baiting and iterative mapping approach. Nucleic Acids Research.

[CR28] Hamari Z, Juhasz A, Kevei F (2002). Role of mobile introns in mitochondrial genome diversity of fungi (a mini review). Acta Microbiologica et Immunologica Hungarica.

[CR29] Hedh J, Johansson T, Tunlid A (2009). Variation in host specificity and gene content in strains from genetically isolated lineages of the ectomycorrhizal fungus Paxillus involutus s. lat. Mycorrhiza.

[CR30] Jargeat P, Chaumeton JP, Navaud O, Vizzini A, Gryta H (2014) The *Paxillus involutus* (Boletales, Paxillaceae) complex in Europe: genetic diversity and morphological description of the new species* Paxillus cuprinus*, typification of* P. involutus* s.s., and synthesis of species boundaries. Fungal Biology 118(1):12–31. 10.1016/j.funbio.2013.10.008 10.1016/j.funbio.2013.10.008 24433674

[CR31] Jargeat P, Moreau PA, Gryta H, Chaumeton JP, Gardes M (2016) *Paxillus rubicundulus *(Boletales, Paxillaceae) and two new alder-specific ectomycorrhizal species, *Paxillus olivellus *and* Paxillus adelphus*, from Europe and North Africa. Fungal Biology 120(5):711–728. 10.1016/j.funbio.2016.02.008 10.1016/j.funbio.2016.02.008 27109368

[CR32] Johri P, Marinov GK, Doak TG, Lynch M (2019) Population genetics of paramecium mitochondrial genomes: recombination, mutation spectrum, and efficacy of selection. Genome Biology and Evolution. 10.1093/gbe/evz081 10.1093/gbe/evz081 PMC6505448 30980669

[CR33] Kanzi AM, Wingfield BD, Steenkamp ET, Naidoo S, van der Merwe NA (2016) Intron derived size polymorphism in the mitochondrial genomes of closely related *Chrysoporthe* species. PLoS One 11(6):e0156104. 10.1371/journal.pone.0156104 10.1371/journal.pone.0156104 PMC4894602 27272523

[CR34] Katoh K, Rozewicki J, Yamada KD (2017) MAFFT online service: multiple sequence alignment, interactive sequence choice and visualization. Briefings in Bioinformatics 20(4):1160-1166.10.1093/bib/bbx108 10.1093/bib/bbx108 PMC6781576 28968734

[CR35] Kazama F, Yatomi Y, Ohmori T, Hosogaya S, Ozaki Y (2001). Elevated expression of mitochondrial NADH dehydrogenase subunit 4/4L genes in vascular endothelial cells undergoing sphingosine-induced apoptosis. Thrombosis and Haemostasis.

[CR36] Kohler A, Kuo A, Nagy LG, Morin E, Barry KW, Buscot F, Canback B, Choi C, Cichocki N, Clum A, Colpaert J, Copeland A, Costa MD, Dore J, Floudas D, Gay G, Girlanda M, Henrissat B, Herrmann S, Hess J, Hogberg N, Johansson T, Khouja HR, LaButti K, Lahrmann U, Levasseur A, Lindquist EA, Lipzen A, Marmeisse R, Martino E, Murat C, Ngan CY, Nehls U, Plett JM, Pringle A, Ohm RA, Perotto S, Peter M, Riley R, Rineau F, Ruytinx J, Salamov A, Shah F, Sun H, Tarkka M, Tritt A, Veneault-Fourrey C, Zuccaro A, Tunlid A, Grigoriev IV, Hibbett DS, Martin F, Mycorrhizal Genomics Initiative C (2015). Convergent losses of decay mechanisms and rapid turnover of symbiosis genes in mycorrhizal mutualists. Nature Genetics.

[CR37] Kurtz S, Choudhuri JV, Ohlebusch E, Schleiermacher C, Stoye J, Giegerich R (2001). REPuter: the manifold applications of repeat analysis on a genomic scale. Nucleic Acids Research.

[CR38] Lanfear R, Frandsen PB, Wright AM, Senfeld T, Calcott B (2017). PartitionFinder 2: new methods for selecting partitioned models of evolution for molecular and morphological phylogenetic analyses. Molecular Biology and Evolution.

[CR39] Lang BF, Gray MW, Burger G (1999). Mitochondrial genome evolution and the origin of eukaryotes. Annual Review of Genetics.

[CR40] Latorre-Pellicer A, Moreno-Loshuertos R, Lechuga-Vieco AV, Sanchez-Cabo F, Torroja C, Acin-Perez R, Calvo E, Aix E, Gonzalez-Guerra A, Logan A, Bernad-Miana ML, Romanos E, Cruz R, Cogliati S, Sobrino B, Carracedo A, Perez-Martos A, Fernandez-Silva P, Ruiz-Cabello J, Murphy MP, Flores I, Vazquez J, Enriquez JA (2016). Mitochondrial and nuclear DNA matching shapes metabolism and healthy ageing. Nature.

[CR41] Lavrov DV, Boore JL, Brown WM (2002). Complete mtDNA sequences of two millipedes suggest a new model for mitochondrial gene rearrangements: duplication and nonrandom loss. Molecular Biology and Evolution.

[CR42] Li H, Shao R, Song N, Song F, Jiang P, Li Z, Cai W (2015). Higher-level phylogeny of paraneopteran insects inferred from mitochondrial genome sequences. Scientific Reports.

[CR43] Li J, Bao S, Zhang Y, Ma X, Mishra-Knyrim M, Sun J, Sa G, Shen X, Polle A, Chen S (2012). Paxillus involutus strains MAJ and NAU mediate K(+)/Na(+) homeostasis in ectomycorrhizal Populus x canescens under sodium chloride stress. Plant Physiology.

[CR44] Li J, Zhang J, Chen H, Chen X, Lan J, Liu C (2013) Complete mitochondrial genome of the medicinal mushroom *Ganoderma lucidum*. PLoS One 8(8):e72038. 10.1371/journal.pone.0072038 10.1371/journal.pone.0072038 PMC3753355 23991034

[CR45] Li Q, Chen C, Xiong C, Jin X, Chen Z, Huang W (2018a) Comparative mitogenomics reveals large-scale gene rearrangements in the mitochondrial genome of two *Pleurotus* species. Applied Microbiology and Biotechnology 102(14):6143-6153.10.1007/s00253-018-9082-6 10.1007/s00253-018-9082-6 29799088

[CR46] Li Q, He X, Ren Y, Xiong C, Jin X, Peng L, Huang W (2020). Comparative mitogenome analysis reveals mitochondrial genome differentiation in ectomycorrhizal and asymbiotic Amanita species. Frontiers in Microbiology.

[CR47] Li Q, Liao M, Yang M, Xiong C, Jin X, Chen Z, Huang W (2018b) Characterization of the mitochondrial genomes of three species in the ectomycorrhizal genus *Cantharellus *and phylogeny of Agaricomycetes. International Journal of Biological Macromolecules 118(Pt a):756-769. 10.1016/j.ijbiomac.2018.06.129 10.1016/j.ijbiomac.2018.06.129 29959010

[CR48] Li Q, Ren Y, Shi X, Peng L, Zhao J, Song Y, Zhao G (2019a) Comparative mitochondrial genome analysis of two ectomycorrhizal fungi (*Rhizopogon*) reveals dynamic changes of intron and phylogenetic relationships of the subphylum Agaricomycotina. International Journal of Molecular Sciences 20(20):5167. 10.3390/ijms20205167 10.3390/ijms20205167 PMC6829451 31635252

[CR49] Li Q, Wang Q, Chen C, Jin X, Chen Z, Xiong C, Li P, Zhao J, Huang W (2018c) Characterization and comparative mitogenomic analysis of six newly sequenced mitochondrial genomes from ectomycorrhizal fungi (*Russula*) and phylogenetic analysis of the Agaricomycetes. International Journal of Biological Macromolecules 119:792–802. 10.1016/j.ijbiomac.2018.07.197 10.1016/j.ijbiomac.2018.07.197 30076929

[CR50] Li Q, Wang Q, Jin X, Chen Z, Xiong C, Li P, Liu Q, Huang W (2019b) Characterization and comparative analysis of six complete mitochondrial genomes from ectomycorrhizal fungi of the *Lactarius* genus and phylogenetic analysis of the Agaricomycetes. International Journal of Biological Macromolecules 121:249–260. 10.1016/j.ijbiomac.2018.10.029 10.1016/j.ijbiomac.2018.10.029 30308282

[CR51] Li Q, Wang Q, Jin X, Chen Z, Xiong C, Li P, Zhao J, Huang W (2019c) Characterization and comparison of the mitochondrial genomes from two* Lyophyllum *fungal species and insights into phylogeny of Agaricomycetes. International Journal of Biological Macromolecules 121:364–372. 10.1016/j.ijbiomac.2018.10.037 10.1016/j.ijbiomac.2018.10.037 30315880

[CR52] Li Q, Wang Q, Jin X, Chen Z, Xiong C, Li P, Zhao J, Huang W (2019d) The first complete mitochondrial genome from the family Hygrophoraceae (*Hygrophorus russula*) by next-generation sequencing and phylogenetic implications. International Journal of Biological Macromolecules 122:1313–1320. 10.1016/j.ijbiomac.2018.09.091 10.1016/j.ijbiomac.2018.09.091 30227210

[CR53] Li Q, Xiang D, Wan Y, Wu Q, Wu X, Ma C, Song Y, Zhao G, Huang W (2019e) The complete mitochondrial genomes of five important medicinal *Ganoderma* species: features, evolution, and phylogeny. International Journal of Biological Macromolecules 139:397–408. 10.1016/j.ijbiomac.2019.08.003 10.1016/j.ijbiomac.2019.08.003 31381907

[CR54] Li Q, Yang L, Xiang D, Wan Y, Wu Q, Huang W, Zhao G (2020) The complete mitochondrial genomes of two model ectomycorrhizal fungi (*Laccaria*): features, intron dynamics and phylogenetic implications. International Journal of Biological Macromolecules 145:974–984. 10.1016/j.ijbiomac.2019.09.188 10.1016/j.ijbiomac.2019.09.188 31669472

[CR55] Li Q, Yang M, Chen C, Xiong C, Jin X, Pu Z, Huang W (2018d) Characterization and phylogenetic analysis of the complete mitochondrial genome of the medicinal fungus* Laetiporus sulphureus*. Scientific Reports 8(1):9104. 10.1038/s41598-018-27489-9 10.1038/s41598-018-27489-9 PMC6002367 29904057

[CR56] Li W, Freudenberg J, Freudenberg J (2019). Alignment-free approaches for predicting novel nuclear mitochondrial segments (NUMTs) in the human genome. Gene.

[CR57] Lin L, Cui P, Qiu Z, Wang M, Yu Y, Wang J, Sun Q, Zhao H (2019). The mitochondrial tRNA (ala) 5587T>C and tRNA (Leu (CUN)) 12280A>G mutations may be associated with hypertension in a Chinese family. Experimental and Therapeutic Medicine.

[CR58] Lohse M, Drechsel O, Kahlau S, Bock R (2013). OrganellarGenomeDRAW--a suite of tools for generating physical maps of plastid and mitochondrial genomes and visualizing expression data sets. Nucleic Acids Research.

[CR59] Losada L, Pakala SB, Fedorova ND, Joardar V, Shabalina SA, Hostetler J, Pakala SM, Zafar N, Thomas E, Rodriguez-Carres M, Dean R, Vilgalys R, Nierman WC, Cubeta MA (2014) Mobile elements and mitochondrial genome expansion in the soil fungus and potato pathogen Rhizoctonia solani AG-3. FEMS Microbiology Letters 352(2):165–173. 10.1111/1574-6968.12387 10.1111/1574-6968.12387 24461055

[CR60] Lowe TM, Chan PP (2016). tRNAscan-SE on-line: integrating search and context for analysis of transfer RNA genes. Nucleic Acids Research.

[CR61] Ma Y, He J, Ma C, Luo J, Li H, Liu T, Polle A, Peng C, Luo ZB (2014) Ectomycorrhizas with *Paxillus involutus* enhance cadmium uptake and tolerance in Populus x canescens. Plant, Cell & Environment 37(3):627–642. 10.1111/pce.12183 10.1111/pce.12183 23937227

[CR62] Nie Y, Wang L, Cai Y, Tao W, Zhang YJ, Huang B (2019) Mitochondrial genome of the entomophthoroid fungus *Conidiobolus heterosporus *provides insights into evolution of basal fungi. Applied Microbiology and Biotechnology 103(3):1379–1391. 10.1007/s00253-018-9549-5 10.1007/s00253-018-9549-5 30569217

[CR63] Park JS, Kim MJ, Jeong SY, Kim SS, Kim I (2016) Complete mitochondrial genomes of two gelechioids, *Mesophleps albilinella* and *Dichomeris ustalella* (Lepidoptera: Gelechiidae), with a description of gene rearrangement in Lepidoptera. Current Genetics 62(4):809–826. 10.1007/s00294-016-0585-3 10.1007/s00294-016-0585-3 26952721

[CR64] Rajashekar B, Samson P, Johansson T, Tunlid A (2007) Evolution of nucleotide sequences and expression patterns of hydrophobin genes in the ectomycorrhizal fungus *Paxillus involutus*. The New Phytologist 174(2):399–411. 10.1111/j.1469-8137.2007.02022.x 10.1111/j.1469-8137.2007.02022.x 17388902

[CR65] Repar J, Warnecke T (2017). Mobile introns shape the genetic diversity of their host genes. Genetics.

[CR66] Rineau F, Roth D, Shah F, Smits M, Johansson T, Canback B, Olsen PB, Persson P, Grell MN, Lindquist E, Grigoriev IV, Lange L, Tunlid A (2012). The ectomycorrhizal fungus Paxillus involutus converts organic matter in plant litter using a trimmed brown-rot mechanism involving Fenton chemistry. Environmental Microbiology.

[CR67] Ronquist F, Teslenko M, van der Mark P, Ayres DL, Darling A, Hohna S, Larget B, Liu L, Suchard MA, Huelsenbeck JP (2012). MrBayes 3.2: efficient Bayesian phylogenetic inference and model choice across a large model space. Systematic Biology.

[CR68] Rozas J, Ferrer-Mata A, Sanchez-DelBarrio JC, Guirao-Rico S, Librado P, Ramos-Onsins SE, Sanchez-Gracia A (2017). DnaSP 6: DNA sequence polymorphism analysis of large data sets. Molecular Biology and Evolution.

[CR69] Salavirta H, Oksanen I, Kuuskeri J, Makela M, Laine P, Paulin L, Lundell T (2014). Mitochondrial genome of Phlebia radiata is the second largest (156 kbp) among fungi and features signs of genome flexibility and recent recombination events. PLoS One.

[CR70] Sandor S, Zhang Y, Xu J (2018). Fungal mitochondrial genomes and genetic polymorphisms. Applied Microbiology and Biotechnology.

[CR71] Sankoff D, Leduc G, Antoine N, Paquin B, Lang BF, Cedergren R (1992). Gene order comparisons for phylogenetic inference: evolution of the mitochondrial genome. Proceedings of the National Academy of Sciences of the United States of America.

[CR72] Schubert M, Lindgreen S, Orlando L (2016). AdapterRemoval v2: rapid adapter trimming, identification, and read merging. BMC Research Notes.

[CR73] Shah F, Rineau F, Canback B, Johansson T, Tunlid A (2013) The molecular components of the extracellular protein-degradation pathways of the ectomycorrhizal fungus *Paxillus involutus*. The New Phytologist 200(3):875–887. 10.1111/nph.12425 10.1111/nph.12425 PMC4282482 23902518

[CR74] Srirattana K, St John JC (2018). Additional mitochondrial DNA influences the interactions between the nuclear and mitochondrial genomes in a bovine embryo model of nuclear transfer. Scientific Reports.

[CR75] Stamatakis A (2014). RAxML version 8: a tool for phylogenetic analysis and post-analysis of large phylogenies. Bioinformatics.

[CR76] Tajima N, Saitoh K, Sato S, Maruyama F, Ichinomiya M, Yoshikawa S, Kurokawa K, Ohta H, Tabata S, Kuwata A, Sato N (2016). Sequencing and analysis of the complete organellar genomes of Parmales, a closely related group to Bacillariophyta (diatoms). Current Genetics.

[CR77] Tambor JH, Ribichich KF, Gomes SL (2008). The mitochondrial view of Blastocladiella emersonii. Gene.

[CR78] Vaidya G, Lohman DL, Meier R (2011). SequenceMatrix: concatenation software for the fast assembly of multi-gene datasets with character set and codon information. Cladistics.

[CR79] Valach M, Burger G, Gray MW, Lang BF (2014). Widespread occurrence of organelle genome-encoded 5S rRNAs including permuted molecules. Nucleic Acids Research.

[CR80] Wang J, Zhang L, Zhang QL, Zhou MQ, Wang XT, Yang XZ, Yuan ML (2017). Comparative mitogenomic analysis of mirid bugs (Hemiptera: Miridae) and evaluation of potential DNA barcoding markers. PeerJ.

[CR81] Wang L, Zhang S, Li JH, Zhang YJ (2018) Mitochondrial genome, comparative analysis and evolutionary insights into the entomopathogenic fungus *Hirsutella thompsonii*. Environmental Microbiology 20(9):3393–3405. 10.1111/1462-2920.14379 10.1111/1462-2920.14379 30117257

[CR82] Xia Y, Zheng Y, Murphy RW, Zeng X (2016) Intraspecific rearrangement of mitochondrial genome suggests the prevalence of the tandem duplication-random loss (TDLR) mechanism in *Quasipaa boulengeri*. BMC Genomics 17(1):965. 10.1186/s12864-016-3309-7 10.1186/s12864-016-3309-7 PMC5122201 27881087

[CR83] Zubaer A, Wai A, Hausner G (2018) The mitochondrial genome of *Endoconidiophora resinifera* is intron rich. Scientific Reports 8(1):17591. 10.1038/s41598-018-35926-y 10.1038/s41598-018-35926-y PMC6279837 30514960

