# Supplementary figures and images for: Comparative mitogenome analysis of two ectomycorrhizal fungi (Paxillus) reveals gene rearrangement, intron dynamics, and phylogeny of basidiomycetes
Source: IMA Fungus. 2020 Jul 2;11:12. doi: 10.1186/s43008-020-00038-8 (PMC7333402; doi:10.1186/s43008-020-00038-8)

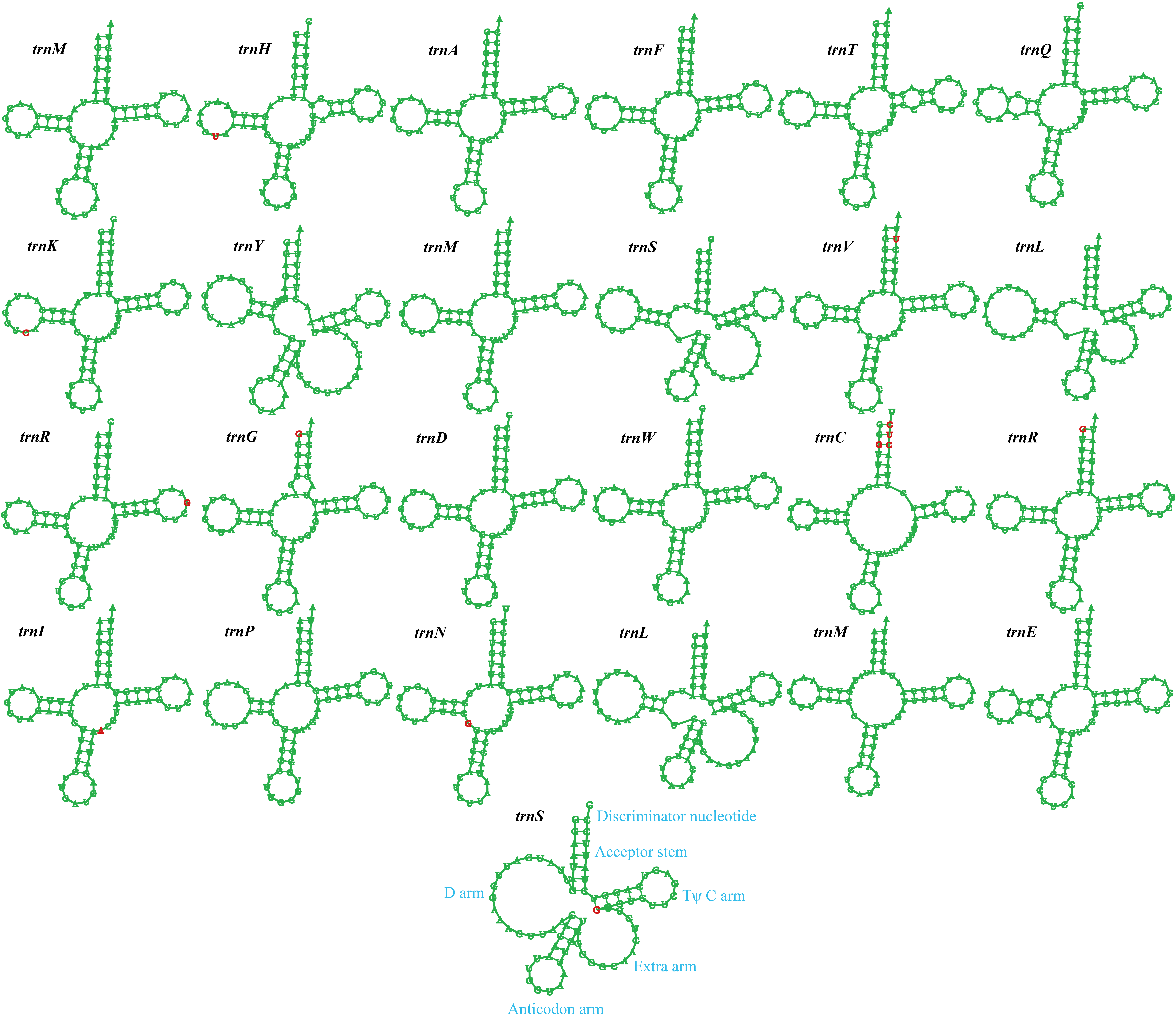

Supplement: Supplementary file 1 — Additional file 1 Fig. S1. Putative secondary structures of the 25 tRNA genes identified in the mitogenomes of two Paxillus species. Residues conserved across the two mitogenomes are shown in green, while variable sites are shown in red. All genes are shown in order of occurrence in the mitogenome of P. involutus, starting from trnM. [file 43008_2020_38_MOESM1_ESM.tif]
